# *Bacillus amyloliquefaciens*: Harnessing Its Potential for Industrial, Medical, and Agricultural Applications—A Comprehensive Review

**DOI:** 10.3390/microorganisms11092215

**Published:** 2023-08-31

**Authors:** Imen Zalila-Kolsi, Afif Ben-Mahmoud, Ray Al-Barazie

**Affiliations:** 1 Faculty of Medical and Health Sciences, Liwa College, Abu Dhabi P.O. Box 41009, United Arab Emirates; ray.albarazie@ect.ac.ae; 2 Neurological Disorders Research Center, Qatar Biomedical Research Institute, Hamad Bin Khalifa University, Doha P.O. Box 34110, Qatar; abenmahmoud@hbku.edu.qa

**Keywords:** *Bacillus amyloliquefaciens*, industrial applications, medical applications, agricultural applications, metabolic engineering

## Abstract

*Bacillus amyloliquefaciens*, a Gram-positive bacterium, has emerged as a versatile microorganism with significant applications in various fields, including industry, medicine, and agriculture. This comprehensive review aims to provide an in-depth understanding of the characteristics, genetic tools, and metabolic capabilities of *B. amyloliquefaciens*, while highlighting its potential as a chassis cell for synthetic biology, metabolic engineering, and protein expression. We discuss the bacterium’s role in the production of chemicals, enzymes, and other industrial bioproducts, as well as its applications in medicine, such as combating infectious diseases and promoting gut health. In agriculture, *B. amyloliquefaciens* has demonstrated potential as a biofertilizer, biocontrol agent, and stress tolerance enhancer for various crops. Despite its numerous promising applications, *B. amyloliquefaciens* remains less studied than its Gram-negative counterpart, *Escherichia coli*. This review emphasizes the need for further research and development of advanced engineering techniques and genetic editing technologies tailored for *B. amyloliquefaciens*, ultimately unlocking its full potential in scientific and industrial contexts.

## 1. Introduction

*Bacillus amyloliquefaciens*, a ubiquitous Gram-positive, aerobic bacterium, is commonly found in soil environments. This versatile organism has been utilized to produce a diverse array of heterologous proteins, including β-glucanases, acid-stable alpha-amylase, mesophilic alpha-amylase, cellulase, acid-soluble proteins, keratinase, and alkaline protease [1,2,3]. These properties make *B. amyloliquefaciens* a valuable host for synthesising therapeutic proteins and industrially relevant enzymes. Furthermore, *B. amyloliquefaciens* offers several advantages for applications in agricultural biotechnology. It produces secondary metabolites that exhibit antimicrobial activities against many phytopathogenic microorganisms, promoting plant growth and enhancing overall plant health [4]. These attributes position *B. amyloliquefaciens* as a promising candidate for developing sustainable and eco-friendly agricultural practices.

*Bacillus amyloliquefaciens* possesses remarkable physiological characteristics and a highly adaptable metabolism, enabling its cultivation of cost-effective media [5]. This bacterium exhibits rapid growth, with a fermentation cycle of approximately 72 h, compared to the 180-h cycle of *Saccharomyces cerevisiae* [6]. *B. amyloliquefaciens* also benefits from robust expression systems with excellent genetic stability and lacks strong codon preferences [7].

*B. amyloliquefaciens* has been established as an industrially significant bacterium in the production of biological indicators for sterilization and in biodefense research [8]. The unique properties and capabilities of this microorganism make it an attractive candidate for various biotechnological applications.

Over the years, extensive research has led to the development of various genetic modification tools for *Bacillus amyloliquefaciens*. These tools range from classical counter-selection marker strategies to the recently developed clustered regularly interspaced short palindromic repeats (CRISPR)—based genetic toolkits. The diverse protein secretion systems, along with the novel artificial promoter and ribosome binding site (RBS) libraries, further facilitate the production of extracellular enzymes [9,10,11,12,13].

*B. amyloliquefaciens* is an ideal multifunctional probiotic, exhibiting significant potential in inhibiting pathogenic bacterial growth and enhancing nutrient assimilation. This microorganism is known to produce a wide array of enzymes, including α-amylase, protease, lipase, cellulase, xylanase, pectinase, aminotransferase, barnase, peroxidase, glucanase, and chitinase [12,14]. BamHI is a type II restriction endonuclease, capable of recognizing short sequences (6 bp) of DNA and specifically cleaving them at a target site [15]. The β-1,3-1,4-glucanase derived from *B. amyloliquefaciens* showed an important role in protecting against phytopathogenic fungi [16].

The inherent genetic background of *B. amyloliquefaciens*, combined with well-developed gene manipulation tools, enables the reconstruction of its cellular metabolism. The availability of public knockout collections further enhances its attractiveness as a host for metabolic engineering applications.

In the agricultural sector, research has demonstrated that adding an appropriate quantity of *Bacillus amyloliquefaciens* can significantly enhance the carbon content in compost, thereby improving soil quality and promoting crop growth [17]. Moreover, *B. amyloliquefaciens* is among the most prevalent bacteria known to colonize plants endophytically, playing a crucial role in the biocontrol of vascular plant pathogens [18].

*B. amyloliquefaciens* is also capable of forming complex biofilms, which can serve as living biological materials to produce various functional biomaterials. These include surface growth factors, antibiotics, lysozyme, and antimicrobial peptides for medical applications. The development of biofilms by *Bacillus amyloliquefaciens* enhances barley’s resistance to salt stress [19]. However, certain bottlenecks currently limit the yield of target heterologous proteins in *B. amyloliquefaciens*, such as the absence of efficient genetic editing systems, unclear transcriptional regulation of heterologous proteins, and restricted secretion of heterologous proteins [20].

Most research has focused on optimizing factors such as signal peptides, transport channel levels, chaperone protein levels, and promoters in the expression and transport systems to enhance heterologous protein production in *B. amyloliquefaciens* [20]. Nevertheless, there is a pressing need to direct research efforts toward designing and developing host chassis to construct sustainable, robust, and efficient microbial cell factories for heterologous protein production.

**Figure 1:**
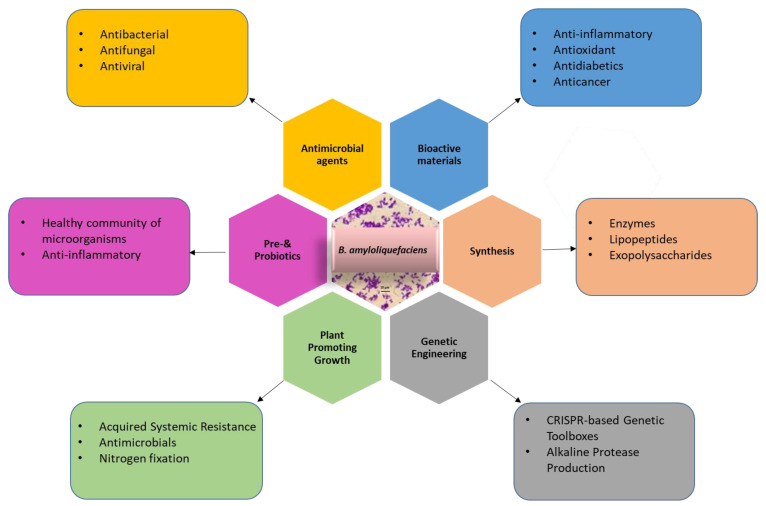
Application of *B. amyloliquefaciens* for genetic engineering, production of industrial chemicals or enzymes, agriculture, medicine, and biomaterials.

This review summarises the latest advancements in metabolic engineering, protein expression systems, and the diverse industrial, medical, and agricultural applications of *Bacillus amyloliquefaciens*. Additionally, we have examined the factors that impede the broader utilization of this microorganism and discussed the underlying reasons. This comprehensive overview serves as a valuable resource for researchers seeking to gain a thorough understanding of *B. amyloliquefaciens* and its myriad applications (Figure 1).

## 2. Genetic Manipulation of *Bacillus amyloliquefaciens*

*Bacillus amyloliquefaciens* is a bacterium of considerable industrial importance, and various genetic manipulation techniques have been employed to enhance its strains for diverse applications [1,8,10,21]. A critical target for genetic modification in *B. amyloliquefaciens* is the alpha-amylase gene (*amyE*), which has been manipulated using innovative plasmid designs for extrachromosomal and intrachromosomal purposes [22].

The *amyE* gene is important because it includes homologous sequences necessary for integration into the *B. amyloliquefaciens* chromosome. This integration can serve as a model for evaluating the success of different genetic engineering strategies: the *amyE* gene’s ability to integrate into the *B. amyloliquefaciens* chromosome can be a practical tool for checking and measuring the performance of various engineering strategies. For instance, if a modification to the *amyE* gene successfully integrates and improves the bacterium’s performance, the applied strategy was effective [22]. Previous research has confirmed the successful application of genetic engineering techniques in *B. amyloliquefaciens* and shown the potential for further optimization and industrial utilization [9,10,13,22,23]. For instance, studies [13,20,23,24,25,26,27] have exhibited enhancements in the production of enzymes, such as protease and lipase, due to genetic engineering. Other research [28,29] has focused on the bacterium’s potential in plant disease control and its role in promoting plant growth. The wide range of applications and the potential for further improvements underline the importance of ongoing research and development.

### 2.1. Development and Application of CRISPR-Based Genetic Toolboxes in Bacillus amyloliquefaciens Strains

Xin et al. (2022) recently reported the development of a CRISPR-based genetic toolkit designed for efficient gene editing, knockout, and integration in *Bacillus amyloliquefaciens* LB1ba02. By employing a single-plasmid CRISPR/Cas9n system, the researchers achieved an impressive 93% knockout efficiency for a single gene and simultaneous editing of three loci with 53.3% efficiency using a base editing CRISPR/Cas9n-AID system [9]. This innovative toolkit was successfully applied to four genes (*aprE*, *nprE*, *wprA*, and *bamHIR*), showcasing its potential as a rapid gene knockout and integration tool for *B. amyloliquefaciens* LB1ba02.

In a unique study, Zhao et al. (2020) developed a novel genetic toolbox to augment the endogenous expression of the mesophilic α-amylase gene in *Bacillus amyloliquefaciens* 205 [13]. A key component of this toolbox was the implementation of an efficient interspecific transformation method, which is a technique that allows genetic material to be transferred across different species of *Bacillus* [30]. This method enhanced the ability to introduce foreign DNA into *B. amyloliquefaciens* 205, thereby facilitating the genetic manipulation of this bacterium. The toolbox also incorporated functional CRISPR systems, which provided precise editing capabilities to further manipulate the genetic material. This innovative strategy, particularly the implementation of interspecific transformation, holds potential applications across a broad range of *Bacillus* species. It allows for the transfer of beneficial traits from one species to another, broadening the possibilities for genetic enhancement in this bacterial genus [30].

The researchers applied several genetic engineering techniques to systematically increase gene expression levels. This included sporulation suppression, a technique that limits the bacteria’s natural process of forming spores, which allows more resources to be dedicated to the expression of the target gene. Additionally, transcript activation techniques were used to increase the production of the specific protein from its corresponding gene, and plasmid-based gene overexpression was utilized to boost the quantity of the target protein being produced. These strategic modifications can be applied across many *Bacillus* species [13].

Furthermore, Chen et al. (2016) successfully promoted spontaneous genetic competence in isolated *B. amyloliquefaciens* strains by overexpressing the master regulator ComK from *Bacillus subtilis* (ComKBsu). Utilizing direct transformation of PCR-generated deletion cassettes, they executed tasks such as replicative plasmid distribution and gene knockout. Artificial induction of genetic competence in *B. amyloliquefaciens* strains carrying the plasmid pUBXC can be achieved through the overexpression of ComKBsu [23].

### 2.2. Enhancing Alkaline Protease Production and Antifungal Properties of Bacillus amyloliquefaciens through Genetic Engineering

In a study focused on enhancing the alkaline protease production capacity of *Bacillus amyloliquefaciens*, researchers employed genetic engineering techniques to improve the production of the alkaline protease BSP-1. They cloned the *bsp-1* gene from a *Bacillus subtilis* strain and introduced it into *B. amyloliquefaciens*. The results demonstrated that the recombinant strain produced a higher quantity of alkaline protease than the wild-type strain, highlighting a potential approach for genetically engineering *B. amyloliquefaciens* to increase the production of novel alkaline proteases [10].

More recent research has directed efforts toward genetically modifying *B. amyloliquefaciens* to enhance its antifungal properties and boost the production of eco-friendly antifungal lipopeptides. The study identified several genetic modifications that improved the engineered strains’ antifungal activity and lipopeptide production. These modifications included the deletion of genes involved in branched-chain amino acid biosynthesis and the overexpression of genes associated with lipopeptide synthesis. These findings suggest that genetic engineering can progressively enhance *B. amyloliquefaciens*’ antifungal capabilities, thereby facilitating the development of sustainable and environmentally friendly antifungal agents [11].

## 3. Gene Expression Using *Bacillus amyloliquefaciens*

**Figure 2:**
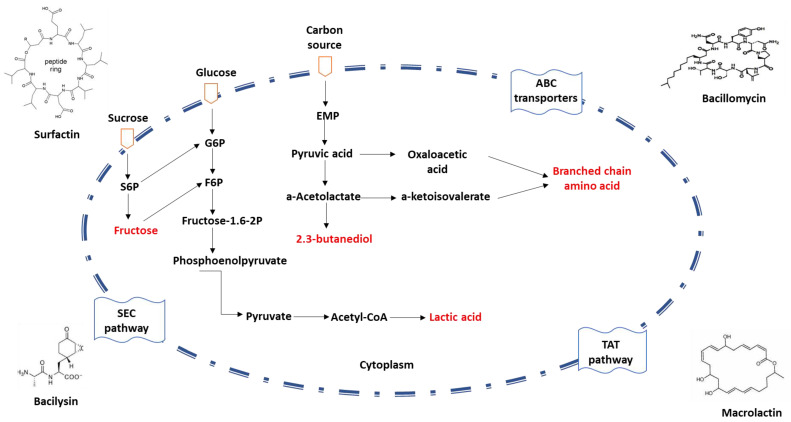
Schematic diagram of protein secretion pathways in *Bacillus* spp. The mechanism of the non-classical secretion pathway is not clear.

Owing to their remarkable capacity to express and secrete proteins, *Bacillus* spp. are frequently utilized in the production of commercial enzyme preparations. To accommodate the secretion requirements of diverse proteins, *Bacillus* spp. possess several promoters and plasmid expression systems. The three conventional protein secretion pathways in *Bacillus* spp. include the general protein secretion pathway (Sec), the twin-arginine translocation pathway (Tat), and the ATP-binding cassette (ABC) transporters [31,32,33] (Figure 2).

### 3.1. Investigating Plant-Bacteria Interaction: Bacillus amyloliquefaciens and Sclerotinia sclerotiorum in Soybean Plants

In a recent investigation, a technique known as digital gene expression profiling was employed to analyze the activity of genes in soybean plants interacting with *Bacillus amyloliquefaciens* and the plant pathogen *Sclerotinia sclerotiorum* [34]. Digital gene expression profiling is a next-generation sequencing method that provides an accurate snapshot of gene activity in a given cell or tissue at a particular time. It allows researchers to identify and quantify specific genes being activated or suppressed in response to various conditions or stimuli.

Using this technique, the study found distinct transcriptional responses in soybean plants interacting with these two organisms. *B. amyloliquefaciens* induced the expression of genes that promote growth and tolerance to stress, while *S. sclerotiorum* stimulated the expression of genes associated with defense and stress responses. This research provides valuable insights into the genetic dynamics of plant interactions with beneficial bacteria and pathogens. Moreover, the study identified several candidate genes differentially expressed in the soybean plants in response to both microorganisms. These ‘candidate genes’ showed significant changes in their activity levels and may play key roles in the plant’s response to *B. amyloliquefaciens* and *S. sclerotiorum*. The study also suggested potential regulatory mechanisms that control the activity of these genes during the plant-bacteria interaction [34].

These regulatory mechanisms can involve various factors and influence how the plant responds to the presence of these microorganisms. This research offers valuable insights into the intricate dynamics between beneficial bacteria, plant pathogens, and their host plants, highlighting how plants can genetically adjust their responses to different microorganisms. Such insights contribute to a deeper understanding of plant-microbe interactions and their implications for crop health and productivity, potentially informing strategies to enhance disease resistance and growth in crops [34].

### 3.2. Cloning and Expression of Bacillus amyloliquefaciens Transglutaminase Gene in E. coli for Food Industry Applications

Duarte et al. (2020) successfully cloned and expressed the transglutaminase (TGase) gene from *Bacillus amyloliquefaciens* in *Escherichia coli* using a bicistronic vector-mediated approach. Transglutaminase enzymes are highly sought after in the food industry due to their ability to enhance food products’ flavor and nutritional value. However, their production has been challenging due to high costs and low yields [25].

To overcome these challenges, the authors constructed a plasmid in which the *B. amyloliquefaciens TGase* gene was fused to the prodomain of the *Streptomyces caniferus* protease. This prodomain is crucial in protein folding, ensuring the TGase enzyme is formed correctly. It also prevents the premature activation of TGase within the bacterial cell, which could disrupt normal cellular processes. To activate the enzyme once it is correctly folded and exported from the cell, the 3C protease gene was also incorporated in the plasmid, allowing for in vivo removal of the prodomain and subsequent activation of the enzyme. The bicistronic vector constructed in this way was then used to transform an *E. coli* strain ready for expression.

The study reported a successful expression of the *B. amyloliquefaciens* TGase gene in *E. coli.* The purified enzyme demonstrated activity across various substrates, indicating potential applications in the food industry [25].

### 3.3. Heterologous Expression and Periplasmic Secretion of an Antifungal Bacillus amyloliquefaciens BLB 369 Endo-β-1,3-1,4-Glucanase in Escherichia coli

**Figure 3:**
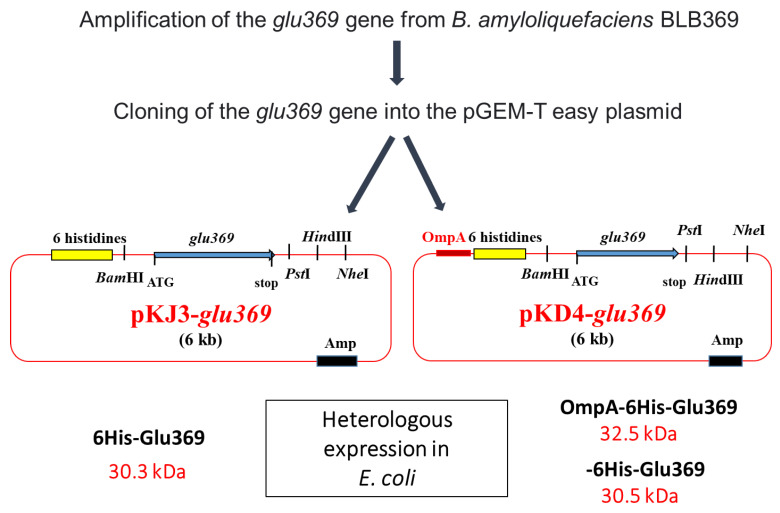
Strategy for cloning the *glu369* gene in pKJ3 and pKJ4 and structure of recombinant plasmids pKJ3-*glu369* and pKD4-*glu369.* Amp, ampicillin resistance; *lacZ*, the gene encoded by the β-galactosidase; OmpA, signal sequence [35].

Endo-β-1,3-1,4-glucanases, classified as glycoside hydrolases, play a crucial role in the enzymatic depolymerization of 1,3-1,4 β-glucans and exhibit antifungal properties. *B. amyloliquefaciens* BLB369 is known to produce this enzyme. Researchers have sequenced, cloned, and effectively expressed the *glu369* full-coding sequence of the endo-β-1,3-1,4-glucanase gene in *Escherichia coli* Top10. To simplify the purification process, the *glu369* coding sequence was integrated into the pKJD4 vector. The resultant OmpA-His-Glu369 fusion protein incorporated the OmpA signal sequence for *E. coli* periplasmic targeting, followed by a 6xHistidine tag for purification purposes. Owing to these beneficial attributes, endo-β-1,3-1,4-glucanase holds significant potential for various biotechnological applications [35] (Figure 3).

To enhance heterologous protein synthesis in *B. amyloliquefaciens*, most existing research has focused on optimizing factors such as signal peptides, transport channel levels, chaperone protein levels, promoters, and other components of the expression and transport systems [20]. Nonetheless, for the establishment of sustainable, dependable, and efficient heterologous protein-producing microbial cell factories, emphasis should be placed on designing and developing host chassis.

Owing to the unique genetic backgrounds of *B. amyloliquefaciens*, several model strains have been extensively investigated. Advanced genome editing techniques have been employed in species like *B. subtilis* and *B. licheniformis* for heterologous protein generation through meticulous microbial chassis engineering [36]. Therefore, it is essential to augment heterologous protein production utilizing *B. amyloliquefaciens*, a strain distinct from *B. subtilis* and *B. licheniformis*. However, our understanding of the regulatory mechanisms governing specific enzyme secretion remains limited. This knowledge gap leads to challenges in devising accurate secretion route engineering strategies and assembling a toolbox of signal peptide sequences for various heterologous enzymes.

Cell membrane engineering is a specialized field that focuses on manipulating the structure and composition of the cell membrane to enhance its functionality. In the case of *Bacillus amyloliquefaciens*, this approach can increase the bacterium’s efficiency in producing and secreting proteins—essentially transforming the bacterium into a ‘cell factory’. However, one of the challenges with this approach lies in predicting the most suitable enzymes to target for a given application. Enzymes are proteins that act as catalysts for various biochemical reactions, and different enzymes are involved in different reactions. Therefore, the choice of enzymes to target can greatly influence the efficiency and effectiveness of protein production and secretion. To address this challenge, one potential strategy is to further optimize cell membrane engineering techniques to be more adaptable to different enzymes. This involves creating a modular system where the techniques can be adjusted and applied predictably depending on the specific enzymes targeted.

In other words, this strategy aims to establish a flexible and standardized framework for cell membrane engineering, where different techniques can be plugged in or taken out as needed to optimize the bacterium’s ability to produce and secrete different target enzymes. This could potentially enhance the usefulness of *B. amyloliquefaciens* in various biotechnological applications.

## 4. The Importance and Applications of the *Bacillus amyloliquefaciens*

*B. amyloliquefaciens* is a versatile and beneficial bacterium with significant importance in various industries due to its ability to produce an array of enzymes and antimicrobial compounds. It is a biocontrol agent in agriculture, promoting plant growth and protecting crops from pathogens. Its ability to produce enzymes such as amylase, protease, and lipase has led to its widespread use in food and beverage, contributing to processes like fermentation, baking, and brewing. Furthermore, *B. amyloliquefaciens* has applications in biotechnology, where it is employed as a host to produce heterologous proteins and as a source of valuable biomolecules. The diverse applications of *B. amyloliquefaciens* highlight its importance in advancing sustainable and eco-friendly solutions across multiple sectors [5,8,11,17,21,24,35,36,37,38,39,40,41,42,43].

### 4.1. Industrial Application of Bacillus amyloliquefaciens

*Bacillus amyloliquefaciens* has shown the ability to produce a variety of amino acids and peptides, which can be relevant to the industrial sector.

γ-aminobutyric acid (GABA), an inhibitory neurotransmitter, has been utilized in treating conditions such as anxiety and sleep disorders. GABA has also shown potential as an anti-aging factor. Although GABA has been used as a food supplement, its efficacy and precise mechanism warrant further research. *Bacillus amyloliquefaciens* EH-9 has been employed for the biosynthesis of GABA, presenting a cost-effective and safe approach. In a study by Zayabaatar et al. (2023), *B. amyloliquefaciens* EH-9 was co-cultivated with germinated rice seeds, and the resulting supernatant was rich in GABA. When this supernatant was applied topically to mice’s dorsal skin, it significantly enhanced type I collagen production. This increase in type I collagen synthesis is attributed to the presence of GABA, as demonstrated by the loss of effect upon knocking down GABA-A receptors in mice [44].

Huang et al., (2023) harnessed *B. amyloliquefaciens* to produce GABA-rich rice by inoculating grain seeds with the bacterium. Mice fed with *B. amyloliquefaciens*-inoculated rice exhibited increased serum levels of neuropeptide Y (Low serum Neuropeptide Y is known to be associated with sleep, circadian rhythm, and emotional disturbances) and improved head dips. A head dip is observed when a mouse puts its head in holes when placed in a hole board test to test anxiety levels. The study suggests this effect is mediated by gastrointestinal GABA-B receptors and the vagus nerve [45].

Poly-γ-glutamic acid (γ-PGA) is a biocompatible and biodegradable polypeptide primarily produced by *Bacillus* species, with diverse applications across various fields [46]. Although *B. amyloliquefaciens* LL3 has demonstrated the ability to synthesize this polypeptide, the yield remains low. Gao et al. (2019) employed genetic and metabolic engineering techniques to enhance the γ-PGA yield in this glutamic acid-independent γ-PGA-producing bacterial strain. In their study, glutamate metabolism pathways were partially inhibited by deleting the *fadR*, *lysC*, *aspB*, *pckA*, *proAB*, *rocG*, and *gudB* genes. The promoter Pc 2up was also inserted into the *icd* gene to regulate NADPH, while the *srf* and *itu* operons were removed. These modifications resulted in the development of a new strain, *B. amyloliquefaciens* NK-A11, which exhibited the highest γ-PGA titer (7.53 g/L), representing a 2.05-fold increase compared to *B. amyloliquefaciens* LL3Δupp [26]. Furthermore, Fang et al. (2020) demonstrated that *B. amyloliquefaciens* JX-6 showed high yields of γ-PGA from fermenting corn stoke and soybean meal, where the yield was 112.82 g/L [47].

Pectinases, enzymes employed in the food industry and other sectors, are typically produced by bacteria or fungi; however, bacterial-derived pectinases are often preferred due to the ease of fermentation and the availability of various techniques to increase yield.

A primary challenge in pectinase production is the associated cost. To address this issue, Doan et al. (2021) utilized *B. amyloliquefaciens* TKU050 to produce pectinases through bioprocessing banana peels, a pectin-containing by-product. By employing banana peels as a carbon source for pectinase synthesis, the approach not only reduced production costs but also promoted environmentally friendly practices. Furthermore, the study suggested that *B. amyloliquefaciens* TKU050 pectinases could have potential applications in prebiotic production [48]. Devaraj et al. (2019) investigated the synthesis of numerous thermostable enzymes by the *B. amyloliquefaciens* strain KUB29. By employing a two-stage fermentation strategy, the researchers identified several enzymes exhibiting activity across various temperatures and pH levels, thereby enhancing enzyme synthesis. These findings indicated that the KUB29 strain holds promise for various industrial biotechnology applications, particularly in biofuel production and other bioproducts, as its enzymes provide a cleaner source than traditional methods [24].

In a recent study, a *B. amyloliquefaciens* D1 strain was isolated from *Morchella crassipes.* This strain can produce high levels of neutral proteases purified using chromatography and ammonium sulfate precipitation. In this study, the purified protease was used in soybean milk fermentation as an example of an industrial application where they increased the release of essential amino acids, which could improve the nutritional value of the fermented soybean milk [49].

In the food industry, multi-drug-resistant foodborne pathogens are a great concern. A study has demonstrated that fengycin produced by *B. amyloliquefaciens* JFL21 showed great antifungal activity and antibacterial activity against foodborne Gram-positive and Gram-negative pathogens. Furthermore, antimicrobial compounds produced by the JFL21 were purified, named Anti-JFL21 and were found to be composed of different lipopeptides, including surfactin, fengycin and iturin. This study has found that Anti-JFL21 showed great antimicrobial activity even to the multi-drug resistant pathogens with low impact on the probiotics [50].

Soussi et al. (2019) conducted a study illustrating that *B. amyloliquefaciens* C5 pro-duced potent antimicrobial surfactant lipopeptides, namely surfactin and bacillomycin D. The growth and antimicrobial activity of this strain were optimized using grape seed flour substrate, which demonstrated optimal performance at 0.2% (*w*/*v*) [51]. Iturin A is another surfactant lipopeptide produced by *B. amyloliquefaciens*, offering diverse applications in the petrochemical, agricultural, and medical sectors. Gao et al. (2022) successfully im-proved the yield of iturin A by enhancing the expression of genes associated with fatty acid biosynthesis, resulting in the development of a new strain, *B. amyloliquefaciens* HZ-ADFTL2. This strain exhibited an increased iturin A yield of 2.96 g/L, representing a 6.59-fold enhancement compared to the original strain, *B. amyloliquefaciens* HZ-12 [52]. In a study by Lu et al. (2016), the synthesis of fengycin, an effective antifungal lipopeptide, by *B. amyloliquefaciens* fmb-60 was enhanced with the addition of fructose to the culture me-dia. A subsequent study revealed that the increased fengycin synthesis in *B. amyloliquefa-ciens* fmb-60 was attributable to the upregulation of transcriptional factor sigmaH and two-component system *degU* and *degQ*, while downregulating the inhibitory regulator AbrB. Additionally, numerous genes involved in amino acid metabolism, fatty acid me-tabolism, and energy metabolism were upregulated or downregulated [53]. Wang et al. (2022) employed a different approach to improve the yield of fengycin and iturin using metabolically engineered *B. amyloliquefaciens*. They utilized *B. amyloliquefaciens* WH1 as a wild-type strain and knocked out several genes, including *bdh* to block the carbon over-flow metabolic pathway, *kinA* to disrupt bacterial sporulation and extend the production period, *dhbF* to enhance the availability of amino acids and fatty acids, and *rapA* to in-crease Spo0A~P levels. The quadruple knockout strain Δ*kinA*Δ*bdh*Δ*dhbF*Δ*rapA* exhibited enhanced antifungal activity, and after fermentation, iturin and fengycin titers in the bio-reactor increased to 123.5 mg/L (a 22-fold increase) and 1200.8 mg/L (a 15.9-fold increase), respectively [11]. Table 1 presents a summary of representative chemicals produced by *B. amyloliquefaciens*.

### 4.2. Medical Application of Bacillus amyloliquefaciens

*B. amyloliquefaciens* SC06 has demonstrated a protective effect against high-fat diet (HFD)-induced obesity in animal models. Wang et al. (2019) found that mice fed an HFD combined with SC06 exhibited reduced fat accumulation, lower obesity levels, and a slight decrease in insulin resistance. Additionally, SC06 treatment provided liver protection and decreased the secretion of serum inflammatory factors, specifically IL-6, TNF-α, and leptin. The study also revealed that SC06 improved the intestinal microbiota composition, contributing to the observed protective effects [55].

Jeong et al. (2020) investigated the impact of *B. amyloliquefaciens* on neuronal activity, microbiome composition, and glucose metabolism in an ischemic stroke model. In their study, Mongolian gerbils were fed chungkookjang, a soybean product fermented with *B. amyloliquefaciens* SRCM100730 (CKJ730) and *B. amyloliquefaciens* SRCM100731 (CKJ731), before inducing ischemic stroke via artery occlusion. The results indicated that the treatment reduced neuronal cell death, ameliorated stroke-related neurological symptoms, enhanced glucose metabolism by preserving β-cells in the pancreas, and improved insulin sensitivity. Furthermore, the treatment exhibited an anti-inflammatory effect, as evidenced by the reduced serum levels of proinflammatory cytokines TNF-α and IL-1β, while preserving the gut microbiome constituents, particularly *Bacteroidia* and *Clostridia* [56].

A novel *Bacillus amyloliquefaciens* strain, X030, has been identified to produce a lipopeptide called bacillomycin Lb, which exhibits anticancer potential against various cancer cell lines [54]. Additionally, bacillomycin Lb has demonstrated antimicrobial activity [57]. A study by Zhou et al. (2022) revealed that using *B. amyloliquefaciens* X030 improved the microbiome composition in grass carp by promoting the presence of probiotics and reducing opportunistic bacteria in the intestine. The strain was also found to produce macrolactin A, an antibacterial agent that collectively enhanced grass carp resistance to certain infections. Furthermore, grass carp fed with *B. amyloliquefaciens* X030 exhibited increased expression of IL-8 (a chemokine), C3 (involved in liver stress tolerance), and IgM, thereby improving the immunity of grass carp against *Aeromonas hydrophila* X040 and *A. veronii* X005 infections [58].

In hospital settings, *Acinetobacter* spp. is a nosocomial pathogen known for forming robust biofilms and exhibiting increasing antibiotic resistance, making the treatment of infections caused by this pathogen challenging. Al-Dulaimi et al., (2021) conducted a preliminary study evaluating the potential use of a probiotic strain, *B. amyloliquefaciens* B-1895 cell-free supernatant (CFS), against *Acinetobacter* spp. isolates from selected clinical cases. CFS’s antimicrobial and anti-biofilm activities were tested alone or in combination with polymyxin E. The study demonstrated that at high concentrations, CFS of *B. amyloliquefaciens* B-1895 inhibited both the growth of *Acinetobacter* spp. and biofilm formation. The combination of polymyxin E and CFS exhibited a synergistic effect, with lower minimum inhibitory concentration (MIC) and minimum biofilm inhibitory concentration (MBIC) values compared to each agent used individually [59].

*B. amyloliquefaciens* also plays a significant role in animal health. In a 2,4,6-trinitrobenzenesulfonic acid (TNBS)-induced colitis model in mice, camel milk enriched with *B. amyloliquefaciens* PBT-3 (BEY) demonstrated beneficial effects [60]. BEY treatment reduced colitis progression and prevented the deterioration of intestinal epithelia in mice by decreasing proinflammatory cytokines such as IL-1β, IL-6, and TNF-α, modulating IL-8 activity, and upregulating the anti-inflammatory cytokine IL-4 [60].

In a comprehensive investigation of subclinical necrotic enteritis in broiler chickens, researchers observed that pretreatment with *Bacillus amyloliquefaciens* BLCC1-0238, either alone or in conjunction with mannan-oligosaccharides (MOS), led to a decrease in mortality rates, reduced intestinal mucosal lesions, and improved growth performance. This outcome appears to be associated with the elevated expression of claudin-3 and peroxisome proliferator-activated receptor-gamma coactivator-1α in both pretreatment groups. Furthermore, the addition of MOS to the bacterial treatment resulted in upregulation of mucin-2 [61]. In a separate study conducted by Morozova et al. (2022), it was demonstrated that feeding Muc2−/− mice, which possess impaired intestinal barriers, an autoclaved diet devoid of *Bacillus* spp. led to an increased incidence of intestinal inflammation due to alterations in the gut microbiome. Additionally, a decline in fertility was observed [62]. Table 2 provides a comprehensive overview of the medical and agricultural applications of *B. amyloliquefaciens*.

### 4.3. Application of Bacillus amyloliquefaciens in Agriculture

**Figure 4:**
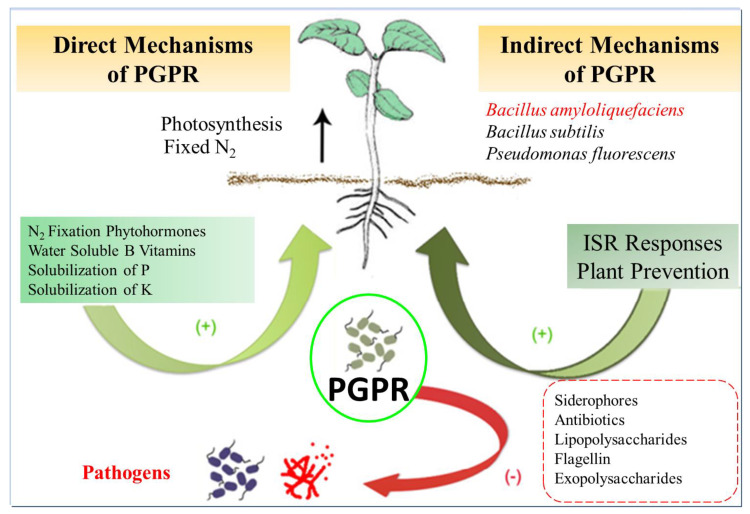
PGPR (Plant growth-promoting rhizobacteria) mechanisms of action. Plant growth-promoting rhizobacteria are microbes associated with plant roots that promote plant growth, supplying improved mineral nutrition, creating hormones or other molecules that stimulate plant growth and strengthen the plant defenses against biotic and abiotic stresses, or defending plants from pathogens by reducing the survival of pathogenic microorganisms. ISR: Induced Systemic Resistance.

*Bacillus amyloliquefaciens*, when isolated from the rhizosphere, has exhibited notable antifungal and antibacterial properties and the ability to promote plant growth, making it a valuable resource in agriculture. Plant growth-promoting rhizobacteria (PGPR) are recognized for their capacity to safeguard plants from biotic and abiotic stress, enhance plant growth, and increase crop yields (Figure 4). The composition of PGPR communities can be influenced by factors such as soil temperature, pH, humidity, and nutrient availability [38]. Rhizobacteria have demonstrated various roles in nutrient acquisition and assimilation, improving soil structure, and producing an array of molecules, including hormones, secondary metabolites, antibiotics, and signaling molecules, all contributing to enhanced plant growth and yield [71]. *B. amyloliquefaciens* has exhibited potential as a biofertilizer, offering a sustainable alternative to conventional synthetic fertilizers [42]. Additionally, the antimicrobial activity of *B. amyloliquefaciens* supports its use in biological control or biocontrol strategies, wherein non-hazardous materials are employed to manage plant diseases, ultimately improving crop yields [43].

*Bacillus amyloliquefaciens*, when utilized as a biofertilizer in conjunction with chemical fertilizers in alkaline farmland soil, has been associated with reduced ammonia volatilization loss, improved crop yields, and enhanced nitrogen recovery. These effects appear to be linked to the inhibition of urease activity and the promotion of potential ammonia oxidation. Moreover, *B. amyloliquefaciens* has been shown to alter the microbial community structure and composition, leading to an increased abundance of ammonia-oxidizing bacteria [42]. In a study involving degraded soil, *Bacillus amyloliquefaciens* B14 was applied to seed inoculation, resulting in improved nutrient availability, enhanced soil enzyme activity, increased microbial respiration, elevated biomass carbon, and altered soil microbial community composition. Collectively, these factors contributed to superior crop growth and yield [17].

*Bacillus amyloliquefaciens* is recognized as a beneficial rhizobacterium for watermelon plants, displaying potential in combating *Fusarium* wilt, a devastating fungal disease caused by *Fusarium oxysporum* f.sp. *niveum* (Fon). Wu et al. (2019) demonstrated that both diffusible and volatile organic compounds (VOCs) produced by *B. amyloliquefaciens* L3 exhibited antifungal activity, particularly against Fon while promoting watermelon growth. The study identified 2-nonanone and 2-heptanone as the most potent antifungal VOCs, with acetoin and 2,3-butanediol acting as growth promoters in a dose-dependent manner [41]. Additionally, Al-Mutar et al. (2023) reported that *B. amyloliquefaciens* DHA55 displayed antifungal activity against Fon and other phytopathogenic fungi, mediated by the production of lipopeptides such as iturin, surfactin, and fungycin [63].

In a separate study, the antifungal effect of *B. amyloliquefaciens* LZN01 against Fon was attributed to the production of myriocin, which disrupts fungal growth at the cellular level. The antifungal mechanism appears to involve myriocin’s impact on the expression of numerous membrane-related genes, primarily affecting sphingolipid metabolism, steroid biosynthesis, and glycerophospholipid metabolism. Additionally, myriocin disturbs protein processing in the endoplasmic reticulum and influences ABC transporters. These effects lead to decreased membrane fluidity and compromised cell membrane integrity, resulting in shrinkage and eventual death of Fon cells [64]. Similarly, *B. amyloliquefaciens* subsp. *plantarum* FZB42 (isolate DMK-7-2) demonstrated antifungal activity against Fon and promoted watermelon growth [29].

Similarly, *Bacillus amyloliquefaciens* B1408 has demonstrated enhanced cucumber growth and biocontrol effects against *Fusarium oxysporum* f.sp. *cucumerinum* (Foc), which causes *Fusarium* wilt in cucumber plants. The beneficial effects of *B. amyloliquefaciens* B1408 appear to be mediated by inducing abnormalities, deformation, and cytoplasmic extravasation in Foc hyphal morphology. Additionally, B1408 alters the rhizosphere microbial community, providing protection against *Fusarium wilt* [65]. In another study, *B. amyloliquefaciens* B9601-Y2 was found to promote maize seedling growth, improve soil quality by enhancing enzyme activity, increase chlorophyll content, and reduce the infection index by antagonizing *Bipolaris maydis*, which causes southern corn leaf blight [37].

Gautam et al. (2019) reported that *B. amyloliquefaciens* S1 exhibits biocontrol effects against *Clavibacter michiganensis*, the causative agent of bacterial canker in tomato plants, when tested under net house conditions. The biocontrol effect is attributed to the production of siderophores, lytic enzymes, and antimicrobial metabolites. Moreover, in vitro experiments demonstrated that *B. amyloliquefaciens* S1 possesses significant phosphorus solubilization and indole acetic acid production capabilities [66]. In a safety assessment,

Anastassiadou et al. (2020) evaluated the use of *B. amyloliquefaciens* AH2 as a fungicide on grapes. The fungicidal effect of *B. amyloliquefaciens* AH2 was deemed effective against grey mold, the target pathogen. Furthermore, the toxicity of *B. amyloliquefaciens* AH2 was assessed in workers handling the bacterium, with no reported side effects or hypersensitivity reactions. However, a few individuals experienced allergic reactions to the bacterium’s metabolites. In mammalian studies, no adverse effects were observed, and the bacterium did not cause infectious disease in rats. However, the toxic effects of the bacterium’s metabolites remain inconclusive, necessitating further assessment. As a result, the study could not exclude the possibility of toxic effects from the bacterium’s products or metabolites [67].

Plant growth-promoting rhizobacteria (PGPR) has been demonstrated to mitigate soil salinization, an abiotic stressor (salt/salinity stress) that negatively impacts crop growth and yield by reducing agricultural land availability [72]. Research has suggested that halotolerant plant growth-promoting bacteria (PGPB), such as *Bacillus amyloliquefaciens* SQR9, are more effective in treating saline soil and restoring balance to enhance crop yield [68]. The potential mechanism underlying the beneficial effects of *B. amyloliquefaciens* SQR9 may involve increased total soluble sugar content, decreased reactive oxygen species, and reduced sodium ion levels, ultimately restoring sodium balance in the soil [73]. Furthermore, *B. amyloliquefaciens* is effective in countering heat stress in plants [69]. In a study examining the effects of *Bacillus amyloliquefaciens* NBRI-SN13 inoculation on rice cv. Saryu-52 seedlings subjected to various abiotic stresses (salt, drought, heat, cold, freezing, and desiccation) and phytohormone treatments, it was found that *B. amyloliquefaciens* NBRI-SN13 promoted proline and total soluble sugar (TSS) production and increased the expression of late embryogenesis abundant (LEA) and dehydrin (DHN) genes, all of which are associated with enhanced stress tolerance. Additionally, SN13 upregulated the expression of glutathione S-transferase (GSTs), an important antioxidant enzyme [70].

Li et al. (2023) raised concerns regarding the toxic effects of polylactic acid microplastic (PLA MPs) exposure on *B. amyloliquefaciens*, which led to oxidative stress, damage to cell wall components, and negative impacts on energy metabolism and cell growth. However, co-exposure to copper ions did not exhibit any synergistic effects. The study demonstrated that the bacterium resisted the toxicity of PLA MPs through sporulation [74].

## 5. Adaptation of *Bacillus amyloliquefaciens* to Acidic Environments: Understanding Acid Tolerance Mechanisms

Recent research has been conducted to investigate the adaptation mechanisms of *Bacillus amyloliquefaciens* in response to acidic environments as defined by the pH scale [75]. The study examined the effects of varying pH levels, notably slight to extremely acidic conditions, on bacterial cell density and their tolerance to low pH stress. The findings revealed that exposure to moderately acidic pH (around 5–6 on the pH scale) enhances the bacterium’s ability to withstand extremely acidic conditions (a pH of 3 or lower), and bacterial cell density plays a crucial role in low pH tolerance. The researchers also identified an acid tolerance response (ATR) mechanism employed by the bacteria to improve their growth under acidic stress. Additionally, the study highlighted the influence of pH levels, as identified on the pH scale, on the bacterium’s gene expression, enabling it to adapt to acid stress. The research provides insights into the molecular pathways through which *B. amyloliquefaciens* adjust to acidic environments [75].

Understanding these adaptation mechanisms may be valuable in developing strategies to mitigate the negative effects of acid stress on bacterial survival and growth, ultimately benefiting various applications of *B. amyloliquefaciens* in agriculture, food production, and environmental management.

## 6. Conclusions and Perspectives

The Gram-positive bacterium *Bacillus amyloliquefaciens* possesses a comprehensive array of genetic tools, promoters, and plasmid expression systems, making it a valuable resource in synthetic biology, metabolic engineering, and protein expression. Furthermore, it can potentially produce chemicals, enzymes, and other industrial bioproducts. This review discussed the advantages of *B. amyloliquefaciens* as a chassis cell in terms of genetic engineering, heterologous gene expression, and its applications in industry, medicine, and agriculture. Despite its numerous promising applications, *B. amyloliquefaciens* remains less studied than its Gram-negative counterpart, *Escherichia coli*, even in the context of advanced methodologies and rapid tool development.

One primary barrier to the widespread adoption of *B. amyloliquefaciens* is its less efficient plasmid synthesis than *E. coli*. To address this issue, future research could focus on developing modified *B. amyloliquefaciens* strains that enable direct plasmid synthesis. Conversely, its high recombination rate offers certain advantages for the development of genome editing tools. *B. amyloliquefaciens* has already been established as a significant platform strain for the commercial production of various chemicals and enzymes. The availability of advanced engineering techniques and cutting-edge genetic editing technologies for controlling and regulating metabolic pathways further enhances the potential of *Bacillus* strains as exceptional industrial production hosts.

This review aimed to provide readers with a comprehensive understanding of the characteristics of *Bacillus amyloliquefaciens*, enabling them to leverage its properties for various applications and research endeavors. The continuous advancement of fundamental and applied studies further solidifies the prominent position of *B. amyloliquefaciens* as one of the leading microbes in the fermentation industry. We anticipate that this evaluation will aid metabolic engineers in effectively harnessing established engineering approaches to guide metabolic engineering efforts for optimizing *B. amyloliquefaciens* cellular factories. Moreover, we hope that increased emphasis will be placed on fostering the development of metabolic engineering strategies and techniques specifically tailored for *B. amyloliquefaciens*.

As more scientists and engineers contribute to research on *B. amyloliquefaciens*, a greater variety of technologies, tools, and methodologies will be employed in the future, enhancing its potential for application in both scientific and industrial contexts.

## Figures and Tables

**Table 1:** Representative chemicals produced by *B. amyloliquefaciens*.

Strains	Used Method	Product	References
*B. amyloliquefaciens* EH-9	Fermentation of Germinated rice seed	GABA	[44]
*B. amyloliquefaciens* NK-A11	Genetic modification of *B. amyloliquefaciens* LL3 a glutamic acid-independent γ-PGA-producing strain	γ-PGA	[26]
*B. amyloliquefaciens* JX-6	Fermentation of corn stalk and soybean milk in sterilize and non-sterilized conditions	γ-PGA	[47]
*B. amyloliquefaciens* TKU050	Bioprocessing of banana peel to reduce production cost	Pectinase/probiotic	[48]
*B. amyloliquefaciens* C5	Growth and antimicrobial activity were optimized using grape seed flour substrate	Surfactin and bacillomycin D	[51]
*B. amyloliquefaciens* HZ-ADFTL2	Genetic modification of *B. amyloliquefaciens* HZ-12 to enhance the yield	Iturin A	[52]
*B. amyloliquefaciens* fmb-60	Addition of fructose to culture media to enhance the yield	Fengycin	[53]
*B. amyloliquefaciens* WH1	Genetic modification was introduced to enhance the yield	Iturin and fengycin	[11]
*B. amyloliquefaciens* X030	Added to various cancer cell lines to test anticancer activity	Bacillomycin Lb	[54]
*B. amyloliquefaciens* JFL21	Tested against multidrug-resistant foodborne pathogens	Multiple lipopeptides	[50]

**Table 2:** List of medical and agricultural applications using *B. amyloliquefaciens*.

Strains	Test Subject	Condition	Bacterial Inoculating Method	Observed Effects	References
*B. amyloliquefaciens* SC06	Mice	High-fat diet (HFD)-induced obesity	Bacteria combined with the HFD fed to the mice	Reduced fat and obesity Mild decrease of insulin resistance Anti-inflammatory Improved intestinal microbiota	[29]
*B. amyloliquefaciens* CKJ730 & CKJ731	Mongolian gerbils	Ischemic stroke model	They were fed chungkookjang, which was fermented using the bacteria	Improved neurological symptoms related to the stroke Increased insulin sensitivity and enhanced glucose metabolism Anti-inflammatory Improved intestinal microbiota	[56]
*B. amyloliquefaciens* X030	Grass carp	Infections	Fed to the grass carps	Enhanced resistance to certain infections Improved intestinal microbiota	[58]
*B. amyloliquefaciens* B-1895	*Acinetobacter* spp. Isolates	*Acinetobacter* growth and biofilm formation	Bacterial cell-free supernatant added to the *Acinetobacter* spp. isolates	Inhibition of the *Acinetobacter* spp. growth Inhibition of biofilm formation	[59]
*B. amyloliquefaciens* PBT-3	Mice	2,4,6-trinitro-benzenesulfonic acid (TNBS)-induced colitis model	Fed camel milk enriched with B. amyloliquefaciens PBT-3 (BEY)	Reduced the progression of the disease and deterioration of the epithelium Anti-inflammatory Up-regulated anti-inflammatory IL-4	[60]
*B. amyloliquefaciens* BLCC1-0238	Broilers	Subclinical necrotic enteritis	Pretreatment with the BLCC1-0238	Reduction of the mortality and intestinal lesions Enhanced growth	[61]
*Bacillus amyloliquefaciens* B14	Degraded soil	Degraded soil	Inoculation of the seeds	Improved nutrient availability, Enhanced soil enzyme activity Increased microbial respiration Altered soil microbial community composition	[17]
*B. amyloliquefaciens* L3 *B. amyloliquefa-ciens* DHA55 *B. amyloliquefaciens* LZN01 *B. amyloliquefaciens subsp. plantarum* FZB42	Watermelon	*Fusarium* wilt caused by *Fusarium oxysporum* f.sp. *niveum (Fon)*	Agricultural use	Biocontrol of fungal disease Promoted growth	[29,41,63,64]
*Bacillus amyloliquefaciens* B1408	Cucumber	*Fusarium* wilt caused by *Fusarium oxysporum* f.sp. *cucumerinum (Foc)*	Agricultural use	Biocontrol of fungal disease Promoted growth	[65]
*B. amyloliquefaciens* B9601-Y2	Maize	southern corn leaf blight caused by *Bipolaris maydis*	Agricultural use	Biocontrol of fungal disease Promoted growth	[37]
*B. amyloliquefaciens* S1	Tomato	Bacterial canker caused by *Clavibacter michiganensis*	Agricultural use	Biocontrol of bacterial disease Promoted growth	[66]
*B. amyloliquefaciens* AH2	Grapes	grey mold	Agricultural use	Biocontrol of the fungus	[67]
*Bacillus amyloliquefa-ciens* SQR9	Saline soil	Saline soil	Agricultural use	Increased total soluble sugar content Decreased reactive oxygen species Reduced sodium ion levels, restoring sodium balance in the soil	[23,68,69]
*Bacillus amyloliquefaciens* NBRI-SN13	rice cv. Saryu-52	Seedlings subjected to various abiotic stresses (salt, drought, heat, cold, freezing, and desiccation) and phytohormone treatments	Agricultural use	Up-regulated the expression of glutathione S-transferase, an antioxidant enzyme Enhanced stress tolerance	[70]

## Data Availability

The original contributions presented in this study are included in the article; further inquiries can be directed to the corresponding author.
